# Performance of Parasitological and Molecular Techniques for the Diagnosis and Surveillance of *Gambiense* Sleeping Sickness

**DOI:** 10.1371/journal.pntd.0002954

**Published:** 2014-06-12

**Authors:** Dieudonné Mumba Ngoyi, Rosine Ali Ekangu, Marie France Mumvemba Kodi, Patient Pati Pyana, Fatima Balharbi, Mélanie Decq, Victor Kande Betu, Wim Van der Veken, Claude Sese, Joris Menten, Philippe Büscher, Veerle Lejon

**Affiliations:** 1 Department of Parasitology, Institut National de Recherche Biomédicale, Kinshasa, Democratic Republic of the Congo; 2 Department of Tropical Medicine, University of Kinshasa, Kinshasa, Democratic Republic of the Congo; 3 Department of Biomedical Sciences, Institute of Tropical Medicine, Antwerp, Belgium; 4 Programme National de Lutte contre la Trypanosomiase Humaine Africaine, Kinshasa, Democratic Republic of the Congo; 5 Department of Clinical Sciences, Institute of Tropical Medicine, Antwerp, Belgium; 6 UMR 177 IRD-CIRAD INTERTRYP, Institut de Recherche pour le Développement, Montpellier, France; Foundation for Innovative New Diagnostics (FIND), Switzerland

## Abstract

**Objectives:**

Recently, improvements have been made to diagnostics for *gambiense* sleeping sickness control but their performance remains poorly documented and may depend on specimen processing prior to examination. In a prospective study in the Democratic Republic of the Congo, we compared the diagnostic performance of several parasite detection techniques, immune trypanolysis and of m18S PCR on whole blood stored in a stabilisation buffer or dried on filter paper.

**Methods:**

Individuals with CATT whole blood (WB) titer ≥1∶4 or with clinical signs indicative for sleeping sickness were examined for presence of trypanosomes in lymph node aspirate (LNA) and/or in blood. Blood was examined with Capillary Centrifugation Technique (CTC), mini-Anion Exchange Centrifugation Technique (mAECT) and mAECT on buffy coat (BC). PCR was performed on whole blood (i) stored in guanidine hydrochloride EDTA (GE) stabilisation buffer and (ii) dried on filter paper, and repeatability and reproducibility were assessed. Immune trypanolysis (TL) was performed on plasma.

**Results:**

A total of 237 persons were included. Among 143 parasitologically confirmed cases, 85.3% had a CATT-WB titre of ≥1/8, 39.2% were positive in LNA, 47.5% in CTC, 80.4% in mAECT-WB, 90.9% in mAECT-BC, 95.1% in TL and up to 89.5% in PCR on GE-stabilised blood. PCR on GE-stabilised blood showed highest repeatability (87.8%) and inter-laboratory reproducibility (86.9%). Of the 94 non-confirmed suspects, respectively 39.4% and 23.4% were TL or PCR positive. Suboptimal specificity of PCR and TL was also suggested by latent class analysis.

**Conclusion:**

The combination of LNA examination with mAECT-BC offered excellent diagnostic sensitivity. For PCR, storage of blood in stabilisation buffer is to be preferred over filter paper. TL as well as PCR are useful for remote diagnosis but are not more sensitive than mAECT-BC. For TL and PCR, the specificity, and thus usefulness for management of non-confirmed suspects remain to be determined.

## Author Summary

Human African trypanosomiasis or sleeping sickness still causes considerable suffering in sub-Sahara Africa. Diagnostics for this infectious disease constantly improve but their performance in terms of accuracy and reproducibility should be evaluated prior to implementation in control activities. We evaluated the diagnostic performance of several microscopic, serological and molecular diagnostic tests on a cohort of 237 sleeping sickness suspects in the Democratic Republic of the Congo. Since molecular diagnostics are rather sophisticated, we also assessed their repeatability and reproducibility. In the absence of a golden standard test, latent class analysis revealed that the suboptimal specificity of the serological and molecular tests is an issue. Our study shows the superior diagnostic sensitivity of the combination of lymph node aspirate examination and separation of trypanosomes from blood by mini Anion Exchange Centrifugation Techniques.

## Introduction

Human African trypanosomiasis (HAT) or sleeping sickness is caused by two subspecies of the protozoan parasite *Trypanosoma brucei (T.b.)* and transmitted through tsetse flies (*Glossina sp*.). *T.b. gambiense* occurs from West to Central sub-Saharan Africa while *T.b. rhodesiense* is endemic in East sub-Saharan Africa. Yearly, between 5,000 and 10,000 sleeping sickness patients are diagnosed and treated [1]. This is only a fraction of those carrying this lethal infection since (i) part of the population at risk lives in remote areas outside the action radius of health centres or mobile teams and (ii) the diagnostic tests suffer from limited sensitivity, and because of (iii) limited participation in active screening and (iv) frequent misdiagnosis in health centres and hospitals [2]–[7]. Developing improved diagnostic tests for sleeping sickness is generally recognised as a high priority [1], [8]. These diagnostic tests should not only be developed for rapid diagnosis, preferably applicable in situations where infrastructure and power supply are basic, but also for monitoring control outcome and for epidemiological surveillance. In the latter case, more sophisticated tests such as the Polymerase Chain Reaction (PCR) or immune trypanolysis (TL) can be applied in a remote reference laboratory [9]. TL is considered as highly specific and has been put forward as a reference test for contact with *T.b. gambiense* [10].

For several decades now, control of *gambiense* sleeping sickness is facilitated by the screening of the population at risk for specific antibodies detectable in the Card Agglutination Test for Trypanosomiasis (CATT) [11]. However, prior to treatment, all CATT positive persons have to undergo parasitological examination. Over the last few years, some improved parasite detection techniques and surrogates for parasite detection have been developed and are proposed for implementation in HAT control and surveillance. The mini-Anion Exchange Centrifugation Technique (mAECT) is the most sensitive method for microscopic trypanosome detection in blood and is based on a purification technique first described by Lanham et al. and later adapted for diagnosis of sleeping sickness [12]–[14]. A new version of the mAECT [15] allows detection of less than 50 trypanosomes/ml. Recently, it has been shown that this threshold can further be lowered by performing the mAECT on the buffy coat obtained by centrifugation of up to 5 ml of blood [16].

PCR-based and similar molecular diagnostics such as Loop-Mediated Isothermal Amplification (LAMP) are considered as highly sensitive and specific [17]–[20]. However, only a few studies provide data on repeatability and reproducibility and on their performance in prospective clinical studies [17], [21]. Furthermore, the outcome of a molecular diagnostic test may be critically influenced by the nature, the volume and the processing of the biological specimens to examine [22], [23]. As reference center for the diagnosis of human African trypanosomiasis, the Unit of Parasite Diagnostics at the Institute of Tropical Medicine recommends two alternative specimen collection and storage protocols for delayed processing in molecular diagnostic tests. In the first protocol, a drop of finger prick blood is dried on a cellulose filter paper and stored in the presence of a desiccant prior to DNA extraction and PCR. In the second protocol, a larger volume (up to 500 µl) of venous blood is mixed with an equal volume of a DNA stabilisation buffer and stored at ambient temperature prior to DNA extraction and PCR.

With the present study we aimed at comparing the diagnostic performance of several parasite detection techniques, of the m18S PCR performed on whole blood stored in a stabilisation buffer and on blood dried on filter paper and of the immune trypanolysis test. For the m18S PCR, repeatability and reproducibility were also investigated.

## Materials and Methods

### Ethics statement

The study received clearance of the Ethics Committee of the University of Antwerp (ITG 09415684) and of the Ministry of Health, DR Congo (M-D/226/2010). Written informed consent of the participants or their guardian was obtained prior to enrolment. In the case of minors, an assent was asked for and parents/guardians provided written informed consent.

### Study design and site

The study was designed as a non-interventional, prospective and cross-sectional study and took place during active screening performed by the mobile team of the Programme National de Lutte contre la Trypanosomiase Humaine Africaine (PNLTHA) in villages of the Masi-Manimba health zone, Bandundu Province, Democratic Republic of the Congo from July 2010 till September 2011. In Bandundu Province, a HAT prevalence of 0.18% was observed within a population of 606,968 examined persons in 2011 [24].

### Study population

The study population consisted of persons recognised as suspect for HAT infection during active screening. Inclusion criteria were: (i) HAT suspicion defined as positivity in CATT at a 1∶4 dilution of blood (equivalent to a 1∶8 dilution of plasma) and/or presence of enlarged lymph nodes (Winterbottom's sign) or other clinical signs suggestive for HAT ; (ii) ≥12 years old; (iii) written informed consent obtained. Exclusion criteria were: (i) previous history of HAT and (ii) less than 7 millilitres of blood obtained. The diagnostic algorithm prescribed by the PNLTHA was followed except that 2 capillaries in stead of 1 capillary of blood were taken. One capillary was used for performing CATT on whole blood. If the CATT test on whole blood was positive, blood from the second capillary was diluted 1∶2–1∶32 and 25 µl of each blood dilution was tested in CATT.

### Specimen collection and parasitological tests

From included suspects with enlarged cervical lymph nodes, a fresh lymph node aspirate (LNA) was examined immediately in bright field microscopy. From all included suspects, 9 ml of venous blood was collected on heparin for sampling for the molecular tests and for the following parasitological tests: (i) Capillary Centrifugation Technique (CTC) [25] with 4 capillaries filled with 60 µl of blood; (iii) mAECT on 350 µl of whole blood (mAECT-WB) as recommended in the instruction leaflet that comes with the test [15]; (iii) mAECT with 500 µl of the buffy coat (mAECT-BC) obtained after low speed centrifugation of 5 ml of blood (1500 g, 10 min) [16]. Two ml of the plasma remaining after centrifugation was collected, frozen and shipped to ITM for immune trypanolysis. All persons that were found positive in parasitological examination underwent lumbar puncture and cerebrospinal fluid (CSF) examination to assess the disease stage. Second stage disease was defined as presence of trypanosomes, assessed by modified single centrifugation, or ≥5 white blood cells/µl in the CSF. In some participants, although no trypanosomes were detected in lymph or blood, the lumbar puncture and CSF examination were carried out on demand of the medical doctor, based on CATT titre and/or on clinical symptoms that alone or in combination were suggestive for sleeping sickness.

### Molecular tests

#### PCR on blood collected on filter paper

On 2 separate Whatman 4 filter paper discs (diameter 9 cm), 4 drops of 30 µl venous blood were spotted and air-dried. Air-dried filter papers were packed individually in paper envelopes and stored in a sealed plastic bag with silicagel at ambient temperature. One filter paper was shipped to INRB, the other one to ITM. Both at ITM and INRB, from each filter, 3 discs of 5 mm diameter were punched out and placed in a 1.5 ml microcentrifuge tube. Between each dried blood specimen, the puncher was cleaned by subsequently dipping in a 1% sodium hypochlorite solution, two times in pure water and finally in pure ethanol whereafter the puncher was air dried. After each series of 10 specimens, discs were punched out from a blank filter to serve as negative extraction control. From these discs, DNA was extracted in duplicate with the QIAamp DNA Micro Kit according to the manufacturer's instruction and a final elution volume of 50 µl AE buffer (Qiagen, Germany). m18S PCR was run in duplicate in a T3 thermocycler 48 (Biometra, Germany) [17]. Amplified products were analysed by electrophoresis in a 2% agarose gel (Eurogentec, Belgium) and UV illumination (Syngene, UK) after ethidium bromide staining of the DNA (Sigma, Belgium).

#### PCR on blood collected in guanidine hydrochloride EDTA buffer (GE-buffer)

Two separate aliquots of 500 µl of venous blood were mixed with 500 µl of GE-buffer (6M guanidine hydrochloride, 0.2 M EDTA, pH 8.0) and gently mixed to complete lysis. Lysed blood was stored at ambient temperature and shipped to respectively INRB and ITM. DNA extraction was performed in duplicate from 400 µl of the blood/GE-buffer mixture with the Maxwell 16 Tissue DNA Purification kit on a Maxwell 16 instrument according to the manufacturer's instructions and a final 300 µl elution volume (Promega, Belgium). Both at INRB and at ITM, m18S PCR was run in duplicate as described above.

Prior to the study, personnel implicated in molecular diagnostics received a training at the WHO Collaborating Centre for Research and Training on Human African Trypanosomiasis Diagnostics in Antwerp, Belgium. This personnel was blinded to the results of the serological and parasitological tests performed at inclusion.

### Immune trypanolysis

Immune trypanolysis was performed according to Van Meirvenne et al [26] with *T.b. gambiense* Variable Antigen Type LiTat 1.3.

### Statistical analysis

All data were entered in Microsoft Excel and subsequently analysed using Stata V12 and R 3.0.1 [27], [28]. To assess the relative sensitivity of the parasitological tests, we compared the number of positives for each test in the whole study population using McNemar's test [29]. We presumed that there were no false positive results and consequently the difference in the number of test positives reflects the differences in sensitivities. Similarly the number of PCR positives was compared between samples collected on filter paper and samples stabilised in GE-buffer. In this analysis, we used the first reading of the filter paper and storage buffer samples, analysed at the ITM. As false positives cannot be excluded for PCR tests (through contamination), we performed a similar analysis as on the mAECT on the PCR test formats, but restricting the analysis to the parasitologically positive cases only.

We also performed Latent Class Analysis (LCA) to estimate the diagnostic accuracy of the diagnostic tests, making use of any positive parasitological result, PCR on samples collected on storage buffer, trypanolysis and CATT≥8 [30]. In the model we presumed that parasitological techniques are per definition 100% specific (i.e. no *Trypanosoma* parasite was wrongly identified as such) and for PCR we used the first reading of the filter paper and storage buffer samples analysed in PCR at the ITM. The model assuming that test results are independent of the infection status of the suspects showed a significant lack of fit. When introducing a correlation between false negative results in parasitology and in PCR, explained by very low parasitemias, the model showed an excellent fit to the data. We also fitted the same model using mAECT on buffy coat or mAECT on whole blood as parasitological tests, using PCR on the filter paper samples, and using alternative cut-off dilutions for CATT.

Finally, we assessed the analytical repeatibility and intralaboratory reproducibility of the PCR tests using filter paper and storage buffer using Kappa statistics. The analytical repeatibility was assessed at the ITM using a single sample run in duplicate at a single instance. The intralaboratory reproducibility was assessed using two separate PCR runs from the same sample using different readers and different reagent batches and was performed in DRC.

### Sample size calculation

The sample size was calculated for comparison of the sensitivity (defined as the percentage of samples tested being positive) between mAECT-WB and mAECT-BC using McNemar's test at the two-sided 5% level and with a power of 90% assuming that the diagnostic sensitivities of mAECT-WB and mAECT-BC are respectively at 70%–85%, and 80%–95% with 20% discordant samples. With these assumptions, the required sample size was 189 samples; to allow for up to 20% processing errors we planned to recruit 230 suspects as defined above. The sample size was calculated based on Lachin [31] as programmed by Acomed Statistik (http://www.acomed-statistik.de/download_engl.html).

## Results

A total of 237 persons, 98 male and 139 female, were included. The age varied between 12 and 76 with an average of 32 years. One hundred four (44.1%) presented enlarged lymph nodes and 165 presented with a variety of signs suggestive for HAT, including headaches, fever, pruritus, somnolence and insomnia, motor disturbances, logorrhoea, neck pain. Two hundred fourteen were positive in CATT at a 1∶4 dilution of blood (median CATT blood dilution titer 8, IQR 4–16). One hundred forty three participants (60.3%) were found positive in one or more parasitological tests. Among these confirmed HAT cases, 50% were in second stage and 48% were male. A summary of all test results is presented in Table 1.

**Table 1:** Number and percentage of positive test results in parasitology, serology and PCR, according to the status of the participants.

		All suspects at inclusion (N = 237)		Non-confirmed suspects (N = 94)		Parasitologically confirmed cases (N = 143)	
		N positive	% positive	N positive	% positive	N positive	% positive
**Any parasitological technique**		**143**	**60.3**	**-**	**-**	**143**	**100**
Lymph node aspirate†		56	23.6	-	-	56	39.2
Capillary tube centrifugation‡		66	28.5	-	-	66	47.5
mAECT-WB		115	48.5	-	-	115	80.4
mAECT-BC		130	54.9	-	-	130	90.9
CSF examination¥ in CCC or MSC		26	11.0	-	-	26	18.2
**Any PCR**		**224**	**94.5**	**87**	**92.6**	**137**	**95.8**
PCR - filter paper							
INRB	duplicate 1	92	38.8	11	11.7	81	56.6
	duplicate 2	157	66.2	47	50.0	110	76.9
ITM	duplicate 1	147	62.0	32	34.0	115	80.4
	duplicate 2	139	58.7	30	31.9	109	76.2
PCR - GE-buffer							
INRB	duplicate 1	146	61.6	21	22.3	125	87.4
	duplicate 2	172	72.6	55	58.5	117	81.8
ITM	duplicate 1	147	62.0	22	23.4	125	87.4
	duplicate 2	146	61.6	18	19.2	128	89.5
**Serology**							
CATT blood titration end titer (≥4)		214	90.3	75	79.8	139	97.2
Trypanolysis (LiTat 1.3)		173	73.0	38	40.4	135	94.4

† Not done in 128 cases as LNA cannot be applied on suspects without swollen lymph nodes (included in the analysis as not positive).

‡ Not done in 5 cases (result missing in the analysis).

CTC can be done on every suspect, therefore, for the denominator the number of suspects examined was taken, assuming that the positivity rate in the 5 missing suspects is not different from the positivity rate in the examined suspects.

¥ Not done in 84 cases as lumbar puncture, for ethical reasons, is not done on all suspects (included in the analysis as not positive).

CCC = cell counting chamber, MSC = modified single centrifugation; mAECT = mini Anion Exchange Centrifugation Technique, BC = buffy coat, WB = whole blood, CSF = cerebrospinal fluid, GE-buffer = guanidine-hydrochloride EDTA buffer.

### Parasitological results

Of the 143 parasitologically confirmed cases, 39.2% (56/143) were positive in lymph node aspirate (LNA), 47.5% (66/139) in CTC, 80.4% (115/143) in mAECT-WB and 90.9% (130/143) in mAECT-BC. Overall, 55.4% (56/101) of the LNA examinations were positive. Within the group of confirmed HAT cases with enlarged lymph nodes, this percentage was 77.8%. Cross-tabulation of the parasitological tests (Table 2) shows that no participant was positive in CTC only and that 6 cases were positive only in lymph node aspirate. Two of them were in first disease stage. In another 6 cases, trypanosomes were only detected in the CSF; all of them had elevated white blood cell numbers (between 63 and 306 cell/µl). The remaining 131 parasitologically confirmed cases (91.6%) were positive using the mAECT on whole blood (115/131, 87.8%) or on buffy coat (130/131, 99.2%).

**Table 2:** Cross table of all combinations of parasitological results excluding those with zero frequency.

LNA	CTC	mAECT-WB	mAECT-BC	CSF	frequency
n	n	n	n	n	94
n	n	n	n	p	6
n	n	n	p	n	11
n	n	n	p	p	2
n	n	p	n	n	1
n	n	p	p	n	21
n	n	p	p	p	11
n	p	p	p	n	32
n	p	p	p	p	3
p	n	n	n	n	6
p	n	n	p	n	3
p	n	p	p	n	14
p	n	p	p	p	2
p	p	p	p	n	29
p	p	p	p	p	2

LNA = lymph node aspirate, CTC = capillary tube centrifugation, mAECT = mini Anion Exchange Centrifugation, WB = whole blood, BC = buffy coat, CSF = cerebrospinal fluid, n = negative (or not done), p = positive, five missing CTC cases included in the tabulation as negative.

From Table 1 it is evident that both mAECT-WB and mAECT-BC were significantly (*p*<0.001) more sensitive than the CTC and LNA examination. There was no significant (*p* = 0.11) difference in sensitivity between LNA examination and CTC parasitology. mAECT-BC was more sensitive than mAECT-WB (*p*<0.001). Sixteen samples were positive in mAECT-BC and not in mAECT-WB; a single sample was positive in mAECT-WB and not in mAECT-BC. The difference in sensitivities was 6.4% (95% CI: 2.6–10.1).

### PCR results

Overall, a high proportion of participants (94.5%) were positive in at least one PCR test (Table 1 and 3). Thirteen participants were negative in all PCR tests/replicates, including one case parasitologically confirmed with trypanosomes in the CSF; this case was also negative in all parasitological tests performed on blood and lymph. On the 143 parasitologically confirmed cases, positivity rates for the 4 replicates of PCR-filter paper (PCR-FP) ranged from 56.6 to 80.4% (81–115/143) and from 81.8 to 89.5% (117–128) for the 4 replicates of PCR-GE buffer. Within the group of confirmed cases, there was no significant difference in positivity rate between PCR-GE and mAECT-BC for 3 out of 4 replicates, while PCR-FP was for all replicates less positive than mAECT-BC (*p*<0.002). Table 3 shows the distribution of participants grouped in function of increasing number of PCR positive results recorded for each participant (maximum = 8). Fifty four samples (from 30 patients in first stage, 23 in second stage and 1 with unknown stage)were positive in all PCR tests/duplicates. The proportion of parasitologically confirmed results increased with an increasing number of PCRs positive: of the 31 samples with one single PCR positive result, only 14.3% were from parasitologically confirmed cases; of the 54 samples with all eight PCRs positive, 98.1% were from parasitologically confirmed cases.

**Table 3:** Tabulation of PCR results. Distribution of participants grouped in function of increasing sum of PCR positive results obtained with their sample in the 8 PCR tests that were performed and proportion of parasitologically confirmed cases in each group.

Sum PCR pos		Filter paper				GE-buffer				
		INRB		ITM		INRB		ITM		
	Participants	A	B	A	B	A	B	A	B	Paras
0		–	–	–	–	–	–	–	–	1 (7.7%)
1		0	7	0	1	3	8	1	1	3 (14.3%)
2	13	2	18	6	2	2	19	2	5	5 (17.9%)
3	21	3	14	9	13	7	19	5	5	7 (28.0%)
4	28	3	8	10	6	6	11	8	8	8 (53.3%)
5	25	6	14	10	10	13	15	16	11	9 (47.4%)
6	15	5	9	24	24	26	21	26	27	24 (88.9%)
7	19	19	33	34	29	35	25	35	35	33 (94.3%)
8	27	54	54	54	54	54	54	54	54	53 (98.1%)

Sum PCR pos: Sum of positive PCR tests per sample; GE-buffer: guanidine hydrochloride EDTA buffer; INRB = Institut National de Recherche Biomédicale; ITM = Institute of Tropical Medicine Antwerp; A and B = replicates A and B on the same sample; Participants: number of participants per group; Paras = parasitologically confirmed sample.

The number of PCR positives was compared between samples collected on filter paper and samples collected in guanidine hydrochloride EDTA buffer (GE-buffer) using the first duplicate results obtained at ITM. There was no significant difference (Table 1, 0%, 95% confidence interval: −5.8–5.8, *p*<0.001) among the two PCR formats. However, when limiting the analysis to parasitologically confirmed cases, a significantly (*p* = 0.02) higher proportion of test positives was obtained with samples collected on GE-buffer (87.4%) than on filter paper (80.4%) (Table 1). The difference was 7.0% (95% confidence interval: 0.1–13.1).

Table 4 shows the analytical repeatability, intra-laboratory and inter-laboratory reproducibility of the PCR tests performed on samples collected on filter paper and in GE-buffer. The analytical repeatability of the PCR on samples collected on filter paper and in GE-buffer was assessed on the results obtained at ITM with each sample, run in duplicate by the same experimenter in the same laboratory environment. Analytical repeatability was modest with 16.9% of the duplicates being differently classified for filter paper samples and 12.2% of the duplicates being differently classified for GE-buffer samples. Analytical repeatability kappa was 0.65 for filter paper samples and 0.74 for GE-buffer samples. The intra-laboratory reproducibility was assessed on the results obtained at INRB with each sample run in duplicate by two experimenters in the same laboratory environment but with two different batches of PCR reagents. Intra-laboratory reproducibility was relatively poor with 30% or more of the duplicates being classified differently. Intra-laboratory reproducibility kappa was 0.46 for filter paper samples and 0.30 for GE-buffer samples. The inter-laboratory reproducibility was assessed on the first test result of each laboratory and was poor for filter paper and modest for GE-buffer samples, with respectively 32.5% and 13.4% of samples differently classified.

**Table 4:** Analytical repeatability and intra- and inter-laboratory reproducibility of the PCR tests.

	Filter paper		GE-buffer	
	Agreement	Kappa	Agreement	Kappa
Analytical repeatability at ITM	83.1%	0.65	87.8%	0.74
Intra-laboratory reproducibility at INRB	70.0%	0.44	68.8%	0.30
Inter laboratory reproducibility†	67.5%	0.38	86.9%	0.72

† first duplicate at ITM and INRB.

ITM = Institute of Tropical Medicine; INRB = Institut National de Recherche Biomédicale; GE-buffer = guanidine hydrochloride EDTA buffer.

### Serological results

**Figure 1:**
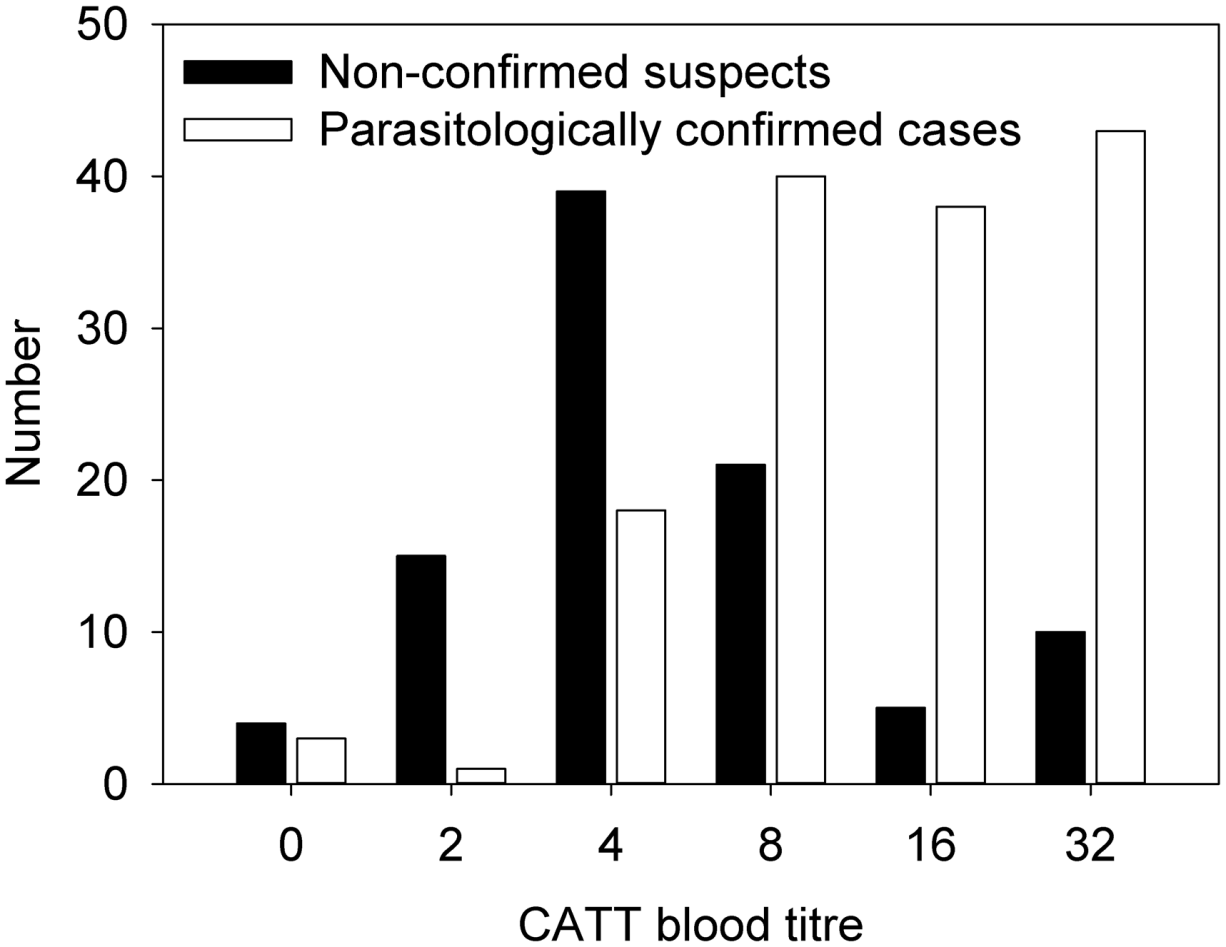
Distribution of CATT end titres in non confirmed suspects and parasitologically confirmed cases.

A summary of the serological results is given in Table 5. Twenty three participants were included with CATT blood titre <4 on the basis of clinical signs. Four (17.3%) of them were confirmed parasitologically. With increasing CATT blood titres, the fraction of parasitologically confirmed cases increased to reach positive predictive values of 77.1%, 84.4% and 81.1% at CATT whole blood titres of respectively ≥8, ≥16 and ≥32 (Figure 1).

**Table 5:** Number and percentage of participants, non confirmed suspects and parasitologically confirmed cases that are immune trypanolysis positive, according to their titre in CATT.

	All suspects at inclusion (N = 237)		Non-confirmed suspects (N = 94)		Parasitologically confirmed cases (N = 143)	
CATT titre	TL positive/n	% TL positive	TL positive/n	% TL positive	TL positive/n	% TL positive
0	2/7	28.6	0/4	0.0	2/3	66.7
2	4/16	25.0	3/15	20.0	1/1	100
4	28/57	49.1	11/39	28.2	17/18	94.4
8	50/61	82.0	11/21	52.4	39/40	97.5
16	41/43	95.3	5/5	100	36/38	94.7
32	48/53	90.6	8/10	80.0	40/43	93.0
Total	173/237	73.0	38/94	40.4	135/143	94.4

TL = trypanolysis; n = number of CATT positives.

Immune trypanolysis was positive in 73% (173/237) of participants and positive predictive value for confirmed HAT was 78.0% (135/173). Overall, the fraction of TL positives increased with increasing CATT titre (Table 5). This was mainly due to an increasing fraction of trypanolysis positives among the non confirmed suspects with increasing CATT titre. The fraction of immune trypanolysis positives in parasitologically confirmed cases was 94.4%, and, for those who were positive in CATT, was independent of the CATT titre.

### Latent class analysis

Compared to the number of parasitologically confirmed cases (60.3%), the estimated number of HAT cases in the study cohort was higher (72.4%) using LCA (Table 6). LCA thus indicated that approximately 29 true cases were missed by the parasitological techniques. The sensitivity of the combined parasitological techniques was estimated at 83%. The estimated sensitivity of mAECT-WB was 67% and of mAECT-BC was 76%. PCR showed a similar sensitivity as the mAECT-BC, but also 22% to 33% of false positives, when performed on specimens collected on storage buffer or on filter paper, respectively. In the study population, selected on the basis of symptoms or positive CATT, trypanolysis had a high sensitivity (94%) and reasonable specificity (83%). The sensitivity and specificity of CATT depended on cut-off used. The sensitivity and corresponding specificity of CATT were 96% and 29% at cut-off titer ≥4, 84% and 80% at cut-off titer ≥8 and 56% and 95% at cut-off titer ≥16.

**Table 6:** Sensitivities and specificities of the different diagnostic tests according to Latent Class Analysis with an estimated 72.4% (95% CI: 65.5, 79.1%) of HAT cases in the study cohort.

	Sensitivity % (95% CI)	Specificity % (95% CI)
Parasitology†	82.5 (75.1, 89.6)	100 (–)
mAECT whole blood	66.9 (58.9, 75.2)	100 (–)
mAECT buffy coat	75.9 (67.8, 84.5)	100 (–)
PCR filter paper	73.4 (75.5, 81.6)	67.0 (50.0, 81.8)
PCR GE buffer†	76.9 (69.9, 83.8)	78.0 (61.8, 91.2)
Trypanolysis†	93.5 (87.4, 97.8)	82.5 (65.3, 96.7)
CATT≥4	96.2 (91.5, 99.4)	29.1 (14.3, 47.0)
CATT≥8†	83.6 (75.0, 91.0)	79.6 (63.6, 93.4)
CATT≥16	56.0 (44.6, 66.7)	94.9 (85.2, 100)

† Tests included in main LCA model.

## Discussion

Overall, 60% of included suspects had parasitologically confirmed HAT but the LCA suggests that about 72% of the suspects were actually HAT cases. This implicates that, according to LCA, the sensitivities of the parasitological tests are lower than their positivity rates calculated on the basis of confirmed HAT cases only. The same trend is observed for the molecular tests. mAECT-BC detected significantly more HAT cases than any other parasitological technique for blood examination. Storage of blood on GE buffer increased the repeatability, inter-laboratory reproducibility and sensitivity of the m18S PCR, resulting in equal sensitivity of PCR-GE as mAECT-BC for detection of HAT. Eighty five percent of HAT cases had a CATT WB titre of ≥1/8 and 94 were positive in immune trypanolysis. Sensitivities calculated by LCA were almost identical with 84% for CATT WB≥1/8 and 94% for immune trypanolysis.

Some limitations are inherent to the set-up of the study. The use of CATT as an inclusion criterion might have biased our study population towards CATT positivity and some CATT negative HAT cases presenting with only mild symptoms might have been missed. Lumbar puncture was not performed systematically in all included suspects. It was restricted to cases that had been parasitologically confirmed in the lymph node aspirate and/or blood and an additional 20 individuals selected by the mobile team based on high CATT titre or clinical signs. Follow-up of serological and clinical suspects has indeed shown that they are more likely to be infected, especially if increased white blood cell counts are encountered in the CSF [32]. Most probably, and suggested by LCA, the unconfirmed suspect group contains a considerable fraction of real HAT cases that might have been detected by repeating the parasitological tests several times. Follow-up of the unconfirmed suspect group has been initiated in order to study the evolution of their HAT status. Finally, the study was not intended to compare costs of the different tests, nor were data concerning costs collected, thus we cannot draw any conclusions about those aspects.

For this study, thick drop examination and direct examination of fresh blood were not considered, as their sensitivity was already demonstrated to be inferior to CTC and mAECT [33]. For CTC our results confirm previous observations, with reported sensitivities for HAT of respectively 56.5% and 48.3% [12], [33]. Only taking into account sensitivity, our results show that there is no added benefit in performing CTC for blood examination if mAECT or mAECT-BC are done, which are significantly more sensitive. LCA confirms that mAECT-BC is more sensitive than mAECT on whole blood but it should be noted that mAECT-BC is more complicated to perform than mAECT. Results with mAECT on whole blood are in line with previously reported sensitivities of 75.3% [33], 84.5% [12] and 78.9% [16], although in the former two studies the more sensitive mAECT-BC was not used and the LCA estimates a 67% sensitivity of mAECT-WB. Compared to the sensitivity of mAECT-BC reported earlier [16], this test detected a slightly lower proportion of parasitologically confirmed cases in our study, which may be caused by the fact that some of our HAT cases were positive in CSF only, while CSF examination was not taken into account in the previous study. Reported sensitivities for trypanosome detection in the lymph node aspirate largely differ between studies and foci, ranging from 18.8% in Bandundu Province in DR Congo [33], 34.3% in Côte d'Ivoire [12] to 77.2% in Guinea [16]. In our hands, lymph node examination was positive in 39.2% of the parasitologically confirmed cases, and picked up 4.2% of these confirmed cases who otherwise would have been missed. Thus, the present study demonstrates that the mere combination of lymph node examination, that is simple and rapid, with mAECT-BC, that is highly sensitive, yielded the highest diagnostic sensitivity (95.1%). Still, a considerable fraction (4.2%) of the parasitologically confirmed cases were detected with trypanosomes in the CSF only.

Compared to filter paper, collection of blood on GE-buffer is to be preferred since it offers higher repeatability, inter-laboratory reproducibility and sensitivity of the m18S PCR, which may be due to the larger volume of blood and the closed tube format. In this study, we collected blood on Whatman 4 filter paper since this allows to use the filter paper not only for DNA extraction and subsequent molecular testing but also for protein elution and subsequent antibody or antigen detection. For example, the immune trypanolysis test that was carried out on plasma in this study can as well be performed on filter paper eluate [34]. Elution of antibodies is not possible with filter papers that are specifically designed for preservation of DNA, like the FTA card. We cannot exclude that the use of FTA cards and other DNA extraction methods might have yielded higher sensitivity than observed in this study [23]. The proportion of parasitologically confirmed cases that were found positive in m18s PCR-GE buffer at INRB and at ITM are in line with the previously reported sensitivity of 88.4% for this PCR in a prospective study on *T.b. gambiense* cases in DR Congo, using a similar sample collection system based on commercially available AS1 buffer [17]. Remote DNA detection does not seem to offer more sensitive diagnosis of HAT than mAECT-BC or the combination of mAECT-BC and lymph node examination, but, taking into account the relatively low specificities (67–78%) calculated with LCA may still be useful for samples collected at more peripheral levels. Repeatability and inter-laboratory reproducibility for the m18S PCR-GE buffer are comparable to the 78.4% repeatability and 86.6% reproducibility of PCR-oligochromatography in trypanosome spiked blood specimens, as observed in a ring trial [21]. Problems of reproducibility of PCR for diagnosis of HAT have been reported earlier [35], especially in aparasitemic serological suspects. This trend seems to be confirmed in the non-confirmed suspects in the present study (data not shown). A possible explanation is to assume that in these persons parasitaemia is below the microscopic detection limit and that the corresponding target DNA concentration is close the PCR detection limit, resulting in low repeatability and reproducibility [21], [35].

Antibody detection by CATT [11] has been used for active HAT screening of high risk populations for over three decades. Importantly, 2.1% of HAT cases were CATT negative in the present study, underlining the significance of clinical signs such as enlarged lymph nodes, headache and fever, in diagnosis of HAT. Subsequent testing of serum or plasma dilutions in CATT is performed to achieve higher specificity before carrying out parasitological examinations without significantly compromising sensitivity, or to assist in deciding on treatment or follow-up of individuals who after parasitological examination remain trypanosome negative [32], [36]. In the present study, we used dilutions of capillary blood for easy manipulation, with a cut-off of ≥1/4, corresponding to a ≥1/8 plasma cut-off.

With increasing CATT blood titres, the positive predictive value for HAT and the number of TL positive suspects increased. The TL test is considered as a highly specific reference test for antibody detection, although LCA suggests that the specificity of TL is only 83%. In West Africa, TL was shown to have 100% sensitivity [10]. Sensitivity seems slightly lower in our study, probably due to regional variations of the variable antigen type repertoire, and is in line with previously reported sensitivities of 97.1% and 95.8% for DR Congo [26], [33]. Due to its technical requirements, immune trypanolysis, like PCR, will remain restricted to remote testing either at population level for surveillance of HAT elimination [37] or at an individual level. It may increasingly be used for samples collected at more peripheral levels, *e.g.* as a reference or confirmation test for antibodies after the local use of rapid diagnostic tests which have been recently introduced for HAT [38], [39]. Furthermore, TL could assist in management of non-confirmed CATT seropositive suspects. Thus, trypanolysis positive unconfirmed suspects should be followed up individually due to the potential risk of developing HAT although self-cured transient infection with *T.b. gambiense* may also occur [40].

We conclude that the combination of lymph node aspirate examination with mAECT on buffy coat yielded excellent diagnostic sensitivity. For PCR, collection of blood in stabilisation buffer is to be preferred over filter paper. Immune trypanolysis as well as PCR may be useful for remote diagnosis but their potential for management of non-confirmed suspects remains to be determined.

## Supporting Information

Checklist S1 STARD checklist.
(DOC)
